# Induced acute hyperglycemia modifies the barrier function of the intestinal epithelium by tissue inflammation and tight junction disruption resulting in hydroelectrolytic secretion in an animal model

**DOI:** 10.1590/1414-431X2024e13309

**Published:** 2024-04-19

**Authors:** F.J.W.S. Siqueira, F.A.P. Rodrigues, S.A. Ribeiro, H.N. Veras, F.C.S. Ferreira, R.C.L. Siqueira, A.A. dos Santos, A. Havt, A.A.M. Lima

**Affiliations:** 1 Departamento de Fisiologia e Farmacologia, Faculdade de Medicina, Universidade Federal do Ceará, Fortaleza, CE, Brasil; 2 Departamento de Educação Física e Esporte, Instituto Federal de Educação, Ciência e Tecnologia do Ceará, Fortaleza, CE, Brasil; 3 Programa de Pós-Graduação em Ciências Médicas, Departamento de Patologia e Medicina Legal, Faculdade de Medicina, Universidade Federal do Ceará, Fortaleza, CE, Brasil

**Keywords:** Diabetic metabolic syndrome, Intestinal epithelium barrier function, Tight junctions, Oxidative damage, Ion and water intestinal transports

## Abstract

Diabetic-metabolic syndrome (MetS-D) has a high prevalence worldwide, in which an association with the rupture of the intestinal epithelium barrier function (IEBF) has been pointed out, but the functional and morphological properties are still not well understood. This study aimed to evaluate the impact of acute hyperglycemia diabetes on intestinal tight junction proteins, metabolic failure, intestinal ion and water transports, and IEBF parameters. Diabetes was induced in male *Rattus norvegicus* (200-310 g) with 0.5 mL of streptozotocin (70 mg/kg). Glycemic and clinical parameters were evaluated every 7 days, and intestinal parameters were evaluated on the 14th day. The MetS-D animals showed a clinical pattern of hyperglycemia, with increases in the area of villi and crypts, lactulose:mannitol ratio, myeloperoxidase (MPO) activity, and intestinal tissue concentrations of malondialdehyde (MDA), but showed a reduction in reduced glutathione (GSH) when these parameters were compared to the control. The MetS-D group had increased secretion of Na^+^, K^+^, Cl^-^, and water compared to the control group in ileal tissue. Furthermore, we observed a reduction in mRNA transcript of claudin-2, claudin-15, and NHE3 and increases of SGLT-1 and ZO-1 in the MetS-D group. These results showed that MetS-D triggered intestinal tissue inflammation, oxidative stress, complex alterations in gene regulatory protein transcriptions of intestinal transporters and tight junctions, damaging the IEBF and causing hydroelectrolyte secretion.

## Introduction

Type 1 diabetes mellitus (T1D) is an autoimmune T cell-mediated disease, in which the pancreatic beta-cell mass is partially or completely destroyed, leading to hypoinsulinemia and hyperglycemia (1). Similarly, type 2 diabetes mellitus (T2DM) is another condition strongly associated with obesity and metabolic syndrome (MetS) (2). MetS has a high prevalence in several continents, ranging from 37 to 48% in some nations, and has a tendency to increase in the coming years, leading to high public health expenditures (3).

Diabetes-induced metabolic abnormalities are alarming and are based on a multifactorial mechanism, whether we consider the etiology, the clinical manifestations, or the complex therapeutic approaches (4). The systemic metabolic dysregulation seems to result from disturbances in several physiological microsystems. The entire gastrointestinal tract is affected by the DM-metabolic disorder (5). Symptoms are usually non-specific, but may be severe, with manifestations of diarrhea and fluid-electrolyte instability. The physiological mechanisms of the symptoms are very complex, are considered serious factors, and have not been studied in detail (6).

Recent studies have pointed to an association between MetS and disruption of intestinal epithelium barrier function (IEBF) (7,8). Located in the epithelium, this barrier has extremely complex and self-regulated mechanisms that are sensitive to small metabolic and pathobiological changes (9).

In the epithelium of the small intestine, tight junction proteins (TJP) orchestrate the dynamics of the intestinal epithelium. The majority of these proteins, including claudin, are a family of 27 proteins (10). Claudin-1, -3, -4, -5, -7, and -8 confer barrier properties and are often found in tight epithelia like the distal regions of the intestinal epithelium. Others, such as claudin-2 and -15, induce channel formation within the tight junctions (TJ) and are mostly expressed in leaky epithelia like the proximal intestine (9).

Leaky gut syndrome is a complex phenomenon associated with functional disruption of the IEBF. Due to several factors, including local inflammation, oxidative disorder, infectious status, energy deficit, reduction of essential micronutrients, alteration of the microbiota, metabolic derangement, and electrochemical dysfunction of the gut epithelium, it contributes etiologically alone or together to increased paracellular permeation of the intestinal epithelium (11). In addition, the functional and morphological properties of the intestinal epithelium are still not fully understood. In this context, the present study investigated the impact of diabetic-MetS (MetS-D) on IEBF, claudin-2 and -15 gene expressions, and metabolic disruption and its influence on intestinal ion and water transport in a T1DM preclinical study.

## Material and Methods

### Animals and ethics committee

*Rattus norvegicus* Wistar rats (∼15 weeks old, n_total_=46) from the Animal House of the Department of Physiology and Pharmacology of the Federal University of Ceará were used. The animals were kept in metabolic cages, under a 12-h light/dark cycle, controlled temperature (22±2°C), and water and feed *ad libitum*. All procedures were carried out in accordance with the rules of the National Council for the Control of Animal Experimentation (CONCEA) and submitted for approval by the Ethics Committee on the Use of Animals (CEUA) of the Federal University of Ceará (protocol No. 55/16).

### Experimental design

The MetS-D model in male *Rattus norvegicus* Wistar rats (n=6-12, 200-310 g) was performed by administering 0.5 mL of streptozotocin (70 mg/kg; Merck KGaA, Germany) diluted in sodium citrate solution (50 mM) intraperitoneally, as described by Wu et al. (10). The entire experimental protocol was conducted in metabolic cages so that the consumption of water and food and urine volume could be evaluated daily. For this purpose, the animals were allocated to the cages three days before the experimental protocol for adaptation. After the adaptation period, the MetS-D group animals received the inducer (streptozotocin) and the control group animals received the vehicle (sodium citrate solution, 0.5 mL). Daily measurements of water and feed consumption, urine elimination, and weight were recorded until the last experimental day. Seven days after streptozotocin treatment, the clinical criteria of polyuria (increased volume of urine voided), polydipsia (increased water consumption), polyphagia (increased feed consumption), weight loss, and blood glucose value (>200 mg/dL, without fasting) were used to determine the inclusion of animals in the MetS-D group. On the 14th day after induction, blood glucose was measured again to exclude animals that had recovered pancreatic activity. Both experimental groups were anesthetized with ketamine hydrochloride (90 mg/kg, *im*) and xylazine hydrochloride (10 mg/kg, *im*). A vertical longitudinal laparotomy was performed and then blood samples were obtained by puncture of the superior vena cava. The intestinal tissue was collected and then stored at -80°C for subsequent analysis of molecular markers or fixed in 10% buffered formalin for histological analysis. All animals were euthanized by exsanguination after sample collection.

### Body composition analysis: bioelectrical impedance

Rats underwent body assessment by polar tetra bioimpedance (ImpediVET^®^, USA) on day 14 (after induction). For this, the mice were anesthetized, as cited in the experimental design section, and placed on a non-conductive surface with the limbs arranged perpendicular to the body and the tail extended distally. Four electrodes (25×12 gauge needles) were inserted in the subdermal region along the dorsal midline. The central electrodes were inserted between the ears and between the hind paws, while the peripheral electrodes were inserted between the eyes and on the tail, as specified by the manufacturer. The needles were attached to the device. Body weight and length were measured between the central electrodes and entered into the equipment. Then, the electric current was turned on, and the resistance and reactance bioimpedance parameters were obtained by a single spectrum from 4 to 1 MHz in a series of 256 points. The device uses a complex impedance plot to determine total body water, extracellular fluid, and intracellular fluid. Fat-free mass, fat mass, and body mass index were calculated using the software (ImpediVET^®^) attached to the device.

### Aminogram

Serum samples were used for amino acid quantification in the control and MetS-D groups. For this, 450 µL of 10% trichloroacetic acid and 50 µL of internal standard (alpha amino butyric acid - AABA) were added to 500 µL of each serum sample. After the vacuum drying process, 100 µL of eluent A was added. Subsequently, a 100 µL sample of the total solution was pipetted and placed on the reading column for the quantification of glutamic acid, glutamine, alanine, citrulline, and arginine in the UltiMate 3000 high performance liquid chromatography equipment (Thermo Fisher Scientific^®^, USA).

### Morphometric analysis

Samples from the initial, medial, and distal portions of the intestine were transversely sectioned and fixed in 10% formaldehyde buffer for 18 h. After the fixation period, the material was dehydrated, embedded in paraffin, cut into 5.0-µm sections on an impact microtome (Polycut S, Leica, Germany), and stained with hematoxylin and eosin (HE). Subsequently, with the aid of an optical microscope coupled to the image acquisition system (Leica, Germany) and ImageJ software version 1.5a (National Institutes of Health, USA), the areas of all villi and corresponding crypts were measured in each slide of the study groups. We analyzed an average of 4 photos per slide at 100× amplification, corresponding to 3 or 4 animals per group.

### IEBF assessment: lactulose and mannitol test

The evaluation of IEBF through analysis of lactulose and mannitol by liquid chromatography is the gold standard for IEBF studies (12). For this, the animals remained three days prior to the protocol on a low-carbohydrate diet (isocaloric G, Rhoster^®^, Brazil), with the intention that the carbohydrates in the diet did not interfere in the determination of the test sugars. On day 14, after an 8 h fast, all animals received 2.0 mL of the solution by gavage, containing 5.0 g of lactulose (Duphar Laboratories, UK) and 1.0 g of mannitol (Henrifarma Chemicals and Pharmaceuticals LTDA, Brazil) dissolved in 20 mL of water. The animals were maintained on the isocaloric G diet during the collection period. Urine samples, preserved in 0.236 mg/mL chlorhexidine (Sigma Chemical Co., USA), were collected for 24 h after administration of the test solution. Sample volumes were recorded and centrifuged at 18,480 *g* for 3 min at 25°C. Then, 50 μL of an internal standard solution (3.6 mM melibiose diluted in 2.9 mL of distilled water) was added to 50 μL of each sample. The solution was centrifuged (10,000 rpm for 3 min at 25°C), and 50 μL was used for sugar determination in the UltiMate 3000 equipment (Thermo Fisher Scientific^®^). Five standard carbohydrate solutions (0.01, 0.03, 0.1, 0.3, and 1 mM) were used to construct standard curves for lactulose and mannitol biomarkers. Standard curves for lactulose and mannitol were run before each experiment, and the sugars in the standard curves and urine samples were determined by high pressure liquid chromatography with pulsed amperometric detection in the UltiMate 3000 high performance liquid chromatography equipment (Thermo Fisher Scientific^®^), as previously described (12).

### Evaluation of oxidative stress and inflammatory markers

Distal intestine samples from the control and MetS-D groups were used to investigate the enzymatic activity of myeloperoxidase (MPO), reduced glutathione (GSH), and malondialdehyde (MDA) levels to investigate inflammation and oxidative stress according to a previous study (13).

For MDA quantification, distal intestine samples were homogenized in 0.15 M potassium chloride solution. Then, 250 µL of the homogenate was added to 1.5 mL of 1% phosphoric acid and 500 µL of 0.6% thiobarbituric acid solution. This solution was placed in a water bath (95-100°C) for 45 min. The mixture was cooled in an ice bath and then 2 mL of n-butanol was added. The solution was vortexed and centrifuged at 226 *g* for 15 min at 4°C. After centrifugation, the organic phase was removed for reading at 535 nm in the Synergy H^1^ equipment (Biotek^®^, USA). Results are reported in nmol/mg tissue.

To measure GSH activity, distal intestine segments were homogenized in ice-cold 0.02 M EDTA solution. Then, 40 µL of the produced homogenate was added to 160 µL of distilled water and 40 µL of 50% trichloroacetic acid. The solution was then centrifuged at 3000 *g* for 15 min at 4°C and 200 µL of the supernatant was added to 400 µL of 0.4 M TRIS buffer (pH=8.9) and 10 µL of 5,5'-dithiobis-2-acid nitrobenzoic acid (0.01 M) in a 96-well plate. The standard curve for GSH (1.56; 3.12; 6.25; 12.5; 25; 50; 100 µg) and the samples were read at 412 nm in the Synergy H^1^ equipment (Biotek^®^). Results are reported in µg/g tissue.

To quantify MPO activity, distal intestine samples were homogenized with 0.5% hexadecitrimethylammonium bromide (HTAB; pH 6.0) in potassium phosphate buffer, in which a 10% homogenate was produced. The homogenate was mixed, cooled for 20 min (-20°C), centrifuged at 4620 *g* for 7 min at 4°C, and 10 µL of the supernatant from each sample was pipetted in duplicate in a 96-well plate. The amount of 200 µL of dianisidine solution (O-dianisine dichloride and 1% H_2_O_2_) was added to the supernatant in each sample. The reaction was measured at 450 nm in the Synergy H^1^ equipment (Biotek^®^). Results are reported in units per milligram (U/mg) of tissue.

### Evaluation of gene transcription of regulatory proteins of tight junctions and intestinal transporters

Gene transcription of the zonula occludens-1 (ZO-1), occludin, claudin-2, claudin-15, peptide transport 1 (PEPT-1), sodium-glucose linked transporter (SGLT-1), sodium-hydrogen exchanger 3 (NHE3), and cystic fibrosis transmembrane conductance regulator (CFTR) was determined by quantitative reverse transcription-polymerase chain reaction (qRT-PCR). The reference gene used for normalization was glyceraldehyde-3-phosphate dehydrogenase (GAPDH). RNA extraction from distal intestinal samples on day 14 was performed according to the RNeasy Lipid Tissue Mini Kit protocol (Qiagen, Germany). The amount of 1 µg of the total isolated RNA was used for cDNA synthesis using the iScript cDNA Synthesis Kit (Bio-Rad, USA), according to the manufacturer's instructions. For the PCR reaction, 10 µL of Syber Green PCR Master Mix (Applied Biosystems, England), 2.0 µL of each primer (0.2 µM), and 1.0 µL of cDNA samples were used, completing with nuclease-free water to a volume end of 20 µL. The sequences and annealing conditions obtained from the National Center for Biotechnology Information (NCBI) website for each investigated gene are described in Supplementary Table S1. The data obtained were based on the values of the threshold cycle, in which the observed fluorescence is 10 times greater than the baseline fluorescence for each qPCR assay. All amplifications were evaluated for the melting curve, performed to ensure the specificity of the amplification and to detect the formation of initiator dimers or any other non-specific product. From the values of the quantitative cycle (Cq/Ct), the relative levels of RNA were calculated according to the 2^-ΔΔCt^ methodology (14).

### Distal intestine perfusion assessment

In order to investigate the effects of MetS-D on intestinal fluid and electrolyte balance, we applied the intestinal perfusion test in the control and MetS-D groups. After the anesthetic procedure with ketamine hydrochloride (90 mg/kg) and xylazine hydrochloride (10 mg/kg), the animals were submitted to a surgical process, in which the small intestine was identified and exposed. In the distal region, a cannulation was inserted about 1.0 cm from the cecum and, from there, 30 cm was measured towards the proximal region, in which a proximal cannulation was inserted. The tissue was carefully washed with PBS solution to eliminate fecal residues and reintroduced into the abdominal cavity, which was closed to prevent moisture loss. After suturing, perfusion was started with a Modified Ringer's solution through the proximal cannula. We also induced secretory diarrhea with cholera toxin (CT, Sigma Chemical Co.) in the distal portion of the control (CTRL+CT) and MetS-D (MetS-D+CT) groups (15). CT is a toxin produced by *Vibrio cholerae* that induces acute secretory diarrhea through the activation of Cl^-^ channels in the crypts and inhibition of Na^+^ and Cl^-^ absorption in the villi (15). We used CT to simulate diarrhea, a common symptom in diabetic patients. Groups with secretory diarrhea were perfused with 1 µg/mL CT for 30 min before perfusion with the test solutions. The test solution (pH 7.4, -37°C) was added with penolphtalein (50 mg/mL) as a non-absorbable marker and infused with a flow of approximately 0.4 mL/min with the aid of a Masterflex C/L 77120-52 infusion pump (Thermo Fisher Scientific). Intestinal perfusion was performed for 100 min, with perfusate collection every 20 min, totaling 5 samples in this period, and 1/3 of the initial dose of anesthesia was reapplied, when necessary, throughout the experiment. The solute collected in the distal cannula was stored at -20°C for spectrophotometry reading. The animals were kept warm (38°C) by incandescent lamp throughout the perfusion to avoid hypothermia. At the end of the experiment, the perfused intestinal segment was removed for weighing and obtaining its wet weight and dry weight, after drying for 72 h at 90°C, for later use in the calculation of water and electrolyte fluxes. After removing the intestinal segments, the animals were sacrificed following the method described above. Sodium, chloride, and potassium concentrations were determined using a 443-flame photometer (Instrumentation Laboratory, USA). Osmolarity was measured using a 5100-steam pressure osmometer (Wescor, USA). The calculation of the perfusate sample density, osmolarity variation, sodium, potassium, and chloride was performed based on Mourad (16). The values obtained for osmolarity variation and electrolyte transport are reported in µOsm/kg and µEq^.^mL^-1.^g^-1^ of perfused dry intestine, respectively.

### Statistical analysis

Parametric data were analyzed by Student's *t*-test. Nonparametric data were analyzed using the Mann-Whitney test or Kruskal Wallis test. The parametric results are reported as means±SEM and the nonparametric results are reported as median and interquartile range (Q1-Q3). All tests were analyzed using the GraphPad Prism^®^ software (USA), and the results were considered significant when P*<*0.05.

## Results

### Behavioral, biochemical, and morphological dysfunction in the MetS-D model

**Figure 1:**
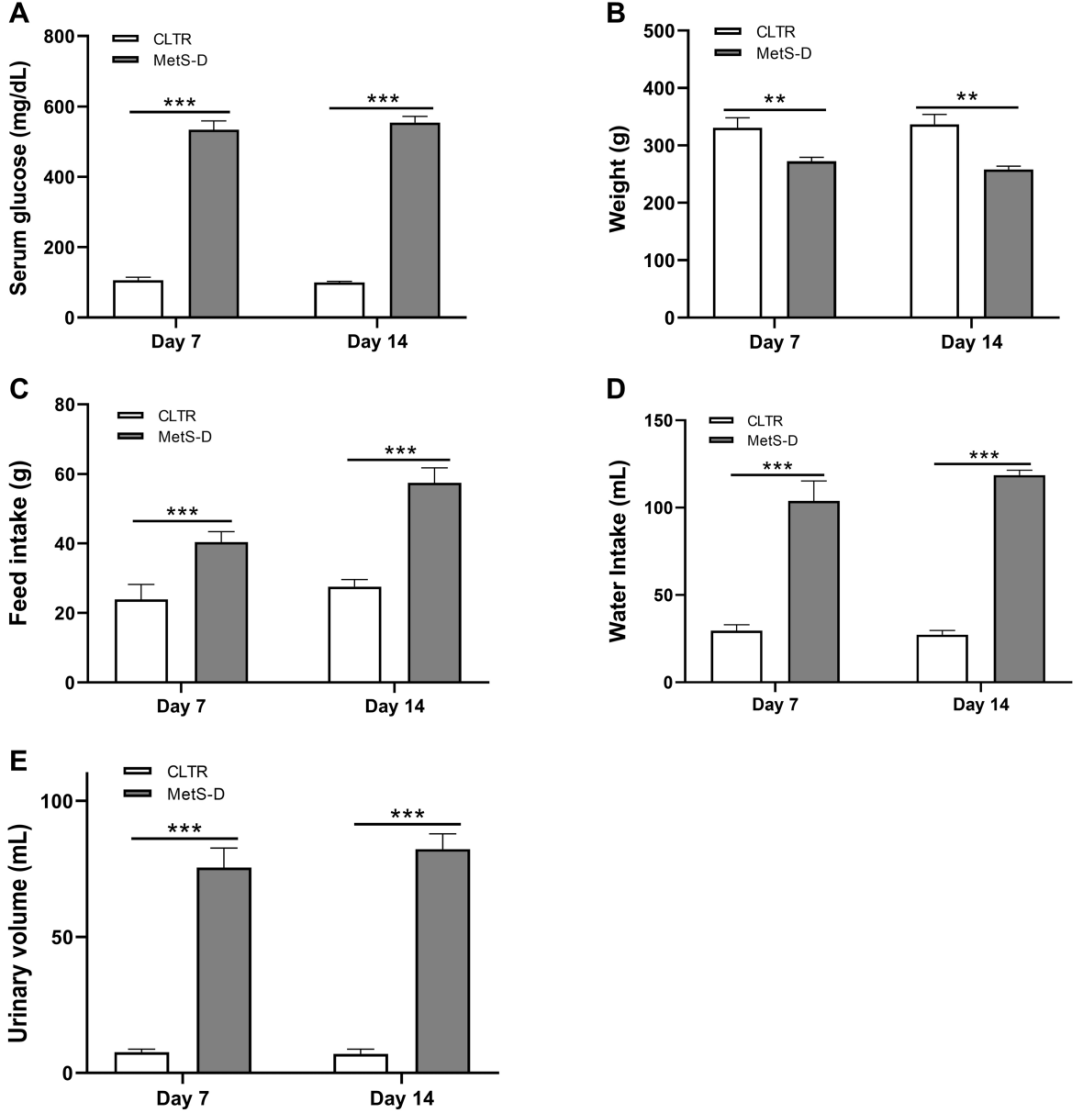
Serum glucose and clinical characterization of the diabetic-metabolic syndrome (MetS-D) animal model and control (CLTR). **A**, serum glucose levels (mg/dL); **B**, body weight (g); **C**, food intake (g); **D**, water intake (mL); and **E**, urine volume (mL) of MetS-D and CLTR animals on days 7 and 14 (n=6-12). Data are reported as means±SEM. **P<0.01, ***P<0.001; Student's *t*-test.

After hyperglycemia induction, the MetS-D group had a higher serum glucose concentration than the control animals (P<0.0001) on day 7 and day 14 (Figure 1A). The body weight of the MetS-D group was also reduced (P<0.01) by 17.67% on day 7 and 23.4% on day 14 compared to the control group (Figure 1B). Both polydipsia and polyphagia were observed in MetS-D animals compared to control animals (P<0.001) (Figure 1C and D). Polyuria was another phenomenon observed in MetS-D animals, characterized by a urinary volume increase of 893% on day 7 and 1074% on day 14 in relation to control animals (Figure 1E).

Metabolic dysfunction in the MetS-D model triggered relevant morphofunctional changes, noted through the reduction of total body water, extracellular fluid, intracellular fluid, fat-free mass, and fat mass compared to the control group on day 14 (P<0.05) (Table 1). However, no changes were found for body mass index. The reduction in total fat-free mass converged with the decrease in plasma albumin, which indicated a negative nutritional profile, and with the nitrogen balance, which was 139% lower in the MetS-D group than in the control group (Control: 377.9±60.66 *vs* MetS-D: -904.4±54.48; P<0.001) (Supplementary Figure S1).

**Table 1:** Body composition of diabetic-metabolic syndrome (MetS-D) and control animals.

Parameters	Control	MetS-D	P values
Total body water (mL)	113.7 (100.2-130.7)	88.69 (83.50-92.28)	0.0051^a^
Extracellular fluid (mL)	113.7 (100.2-130.7)	89.19 (87.52-95.07)	0.0058^b^
Intracellular fluid (mL)	99.8 (93.07-125.1)	88.33 (74.19-93.74)	0.0177^a^
Fat-free mass (g)	105.4 (96.16-127.1)	88.70 (83.50-92.28)	0.0051^a^
Fat mass (g)	113.1 (105.9-134.5)	92.24 (81.21-103)	0.0049^b^
Body mass index (g/cm^2^)	100.3 (90.06-112.4)	96.37 (84.6-98.29)	0.2020^a^

Data are from day 14 of the experiment and are reported as median and interquartile range (Q1-Q3). Medians were compared using the ^a^Mann-Whitney test or ^b^Student's *t*-test.

### Aminogram status in MetS-D animals

**Figure 2:**
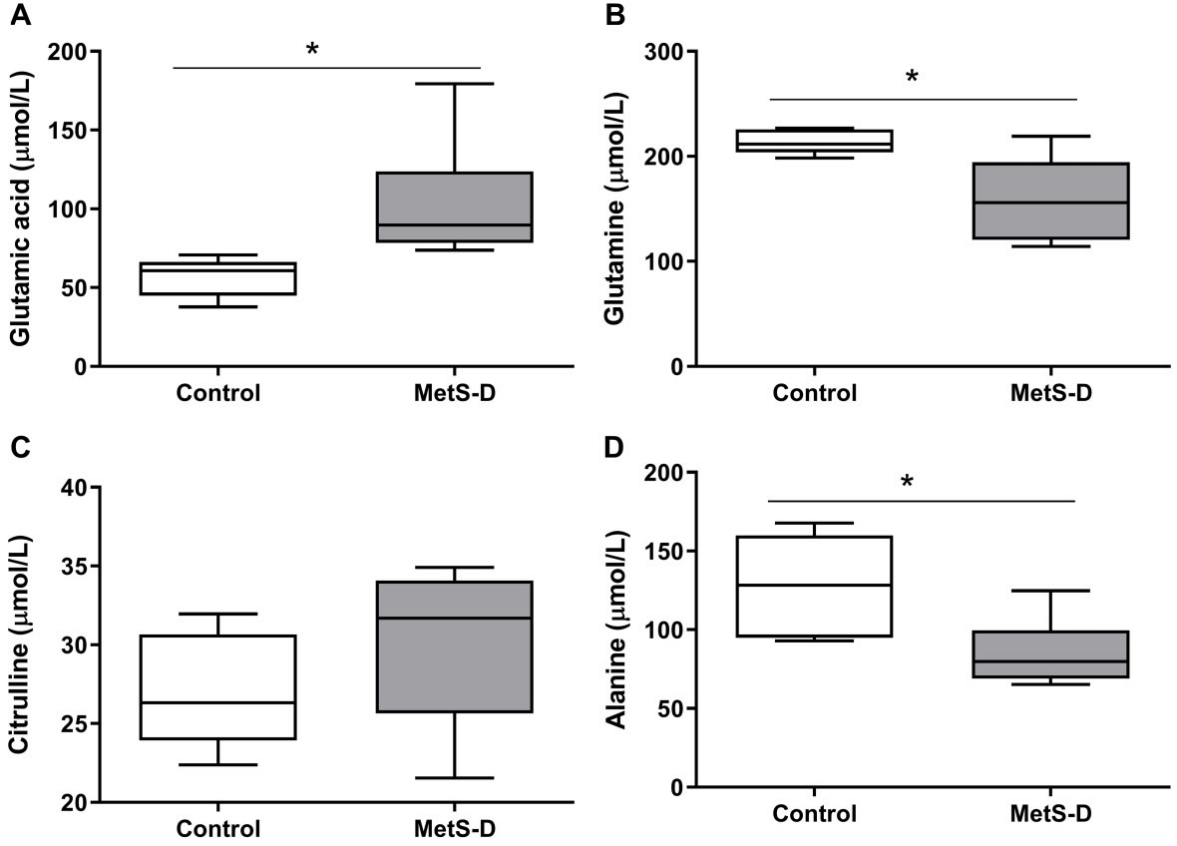
Amino acid serum concentration in diabetic-metabolic syndrome (MetS-D) and control animals. **A**, glutamic acid (µmol/L); **B**, glutamine (µmol/L); **C**, citrulline (µmol/L); and **D**, alanine (µmol/L). Data are representative of day 14 of the experiment. Data are reported as median and interquartile range (Q1-Q3) (n=6-7). *P<0.05, control group *vs* MetS-D group. Student's *t*-test was used for analysis of glutamine, citrulline, and alanine; Mann-Whitney test was used for glutamic acid.

MetS-D animals showed a significant increase in plasma glutamic acid concentration compared to control animals (P<0.05; Figure 2A). However, glutamine and alanine concentrations were significantly reduced in MetS-D animals (P<0.05; Figure 2B and D). Plasma citrulline did not change (P>0.05; Figure 2C)

### MetS-D critically affected intestinal morphometry

**Figure 3:**
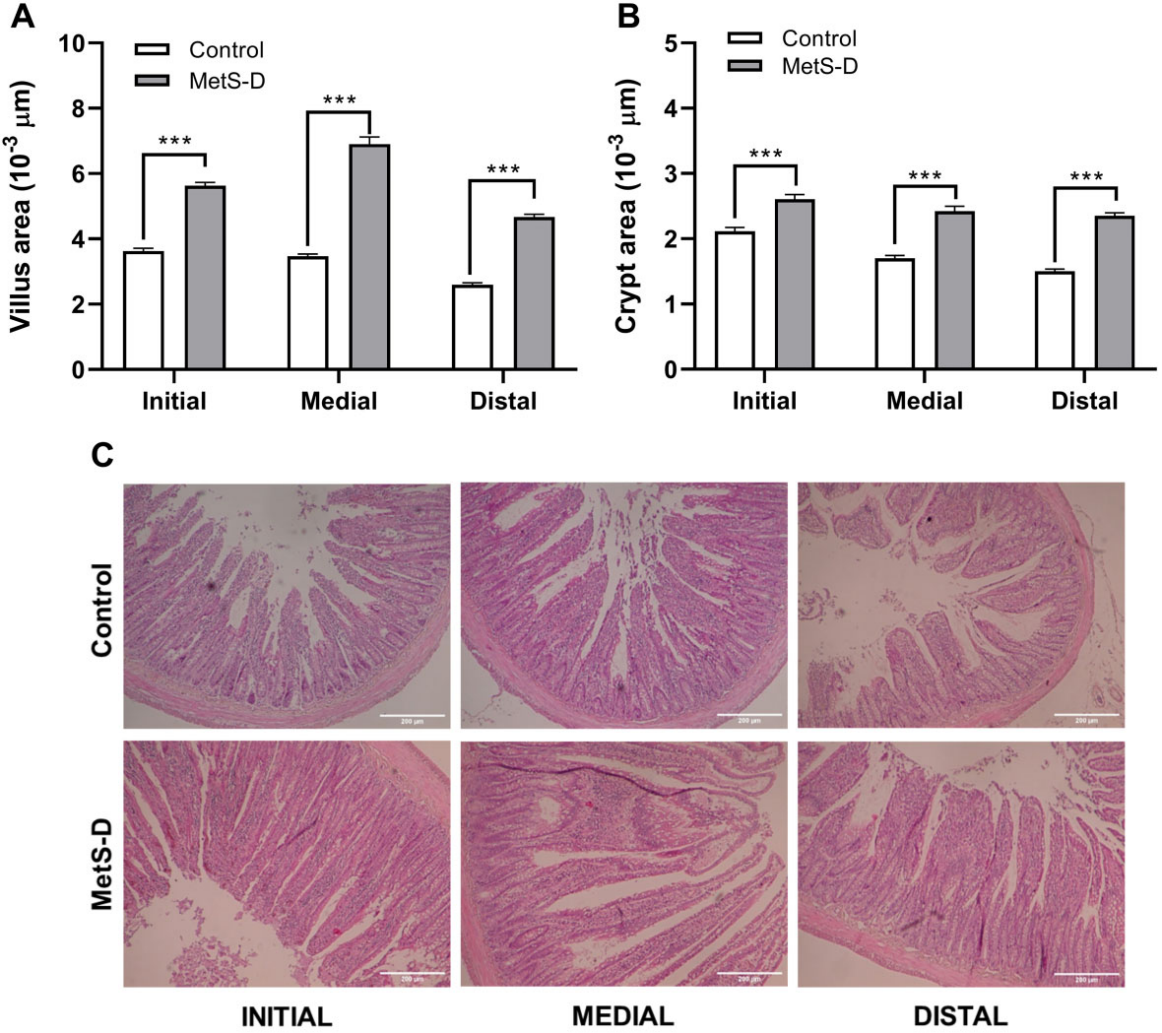
Morphometric analysis of the duodenum (DUO), jejunum (JEJ), and ileum (ILE) of control rats and animals with diabetic-metabolic syndrome (MetS-D). **A**, villus area (10^3^ µm); **B**, crypt area (10^3^ µm); and **C**, photomicrographs of the intestine sections of animals from the MetS-D and control groups. Data are representative of day 14 of the experiment (n=6). 200× magnification (scale bar 100 μm). Data are reported as means±SEM ***P<0.001, Student's *t*-test.

Hyperglycemia induced significant changes in intestinal morphology (Figure 3A, B, and C). There was a significant increase in villi area in the duodenum, jejunum, and ileum of MetS-D animals compared to controls (P<0.001; Figure 3A). Diabetic metabolic dysfunction increased the area of villi in the duodenal, jejunum, and ileum by 55.1, 98.73, and 80.09%, respectively. The crypt areas were also larger in MetS-D animals in the duodenum, jejunum, and ileum (Figure 3B) by 23.32, 42.47, and 56.52%, respectively, compared to the control animals. Representative figures of the duodenum, jejunum, and ileum segments of the MetS-D and control groups are shown in Figure 3C.

### MetS-D dysfunction caused oxidative stress and inflammation in the small intestine

**Figure 4:**
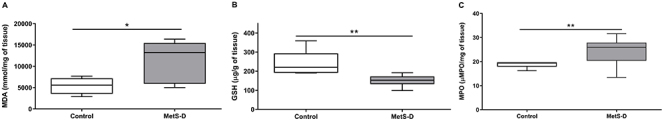
Effects of diabetic-metabolic syndrome (MetS-D) on parameters of oxidative stress and inflammation biomarker in the intestinal tissue. Levels of (**A**) malondialdehyde (MDA), (**B**) reduced glutathione (GSH), and (**C**) myeloperoxidase (MPO) in the ileum of MetS-D and control group animals (n=6-10). The data are representative of day 14 of the experiment. Data are reported as median and Q1-Q3. *P<0.05, **P<0.01, Student's *t*-test for MDA and Mann-Whitney test for GSH and MPO.

MetS-D animals showed a significant increase in MDA levels (P<0.05; Figure 4A). On the other hand, a significant reduction in GSH concentrations was observed in MetS-D animals (P<0.01; Figure 4B). In these animals, inflammation was detected by increased MPO activity in the intestinal tissue (P<0.01; Figure 4C).

### MetS-D animals had leaky gut syndrome

**Figure 5:**
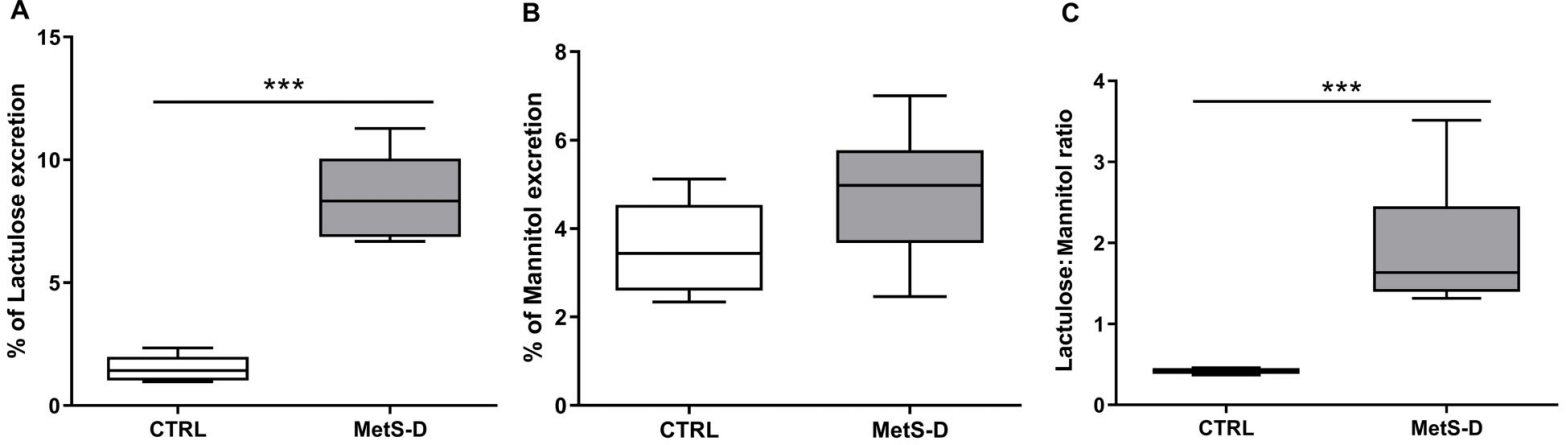
Evaluation of the intestinal epithelium barrier function (IEBF) of diabetic-metabolic syndrome (MetS-D) and control animals (CTRL). **A**, percent of lactulose excretion; **B**, percent of mannitol excretion; and **C**, lactulose:mannitol ratio of MetS-D and CTRL animals (n=6-10). Data are representative of day 14 of the experiment and are reported as median and Q1-Q3. ***P<0.001, Mann-Whitney test.

MetS-D animals had a higher incidence of leaky gut syndrome, detected by critically elevated lactulose excretion (Control 1.51±0.22 *vs* MetS-D 8.5±0.52%) (P<0.001; Figure 5A). Although no significant change was observed in the mannitol excretion rate (Figure 5B), there was a significant increase in the lactulose:mannitol ratio (P<0.001; Control 0.42±0.01 *vs* MetS-D 1.91±0.22), indicating a damage in the IEBF (Figure 5C).

### Hydroelectrolytic analysis of MetS-D in small intestine

**Figure 6:**
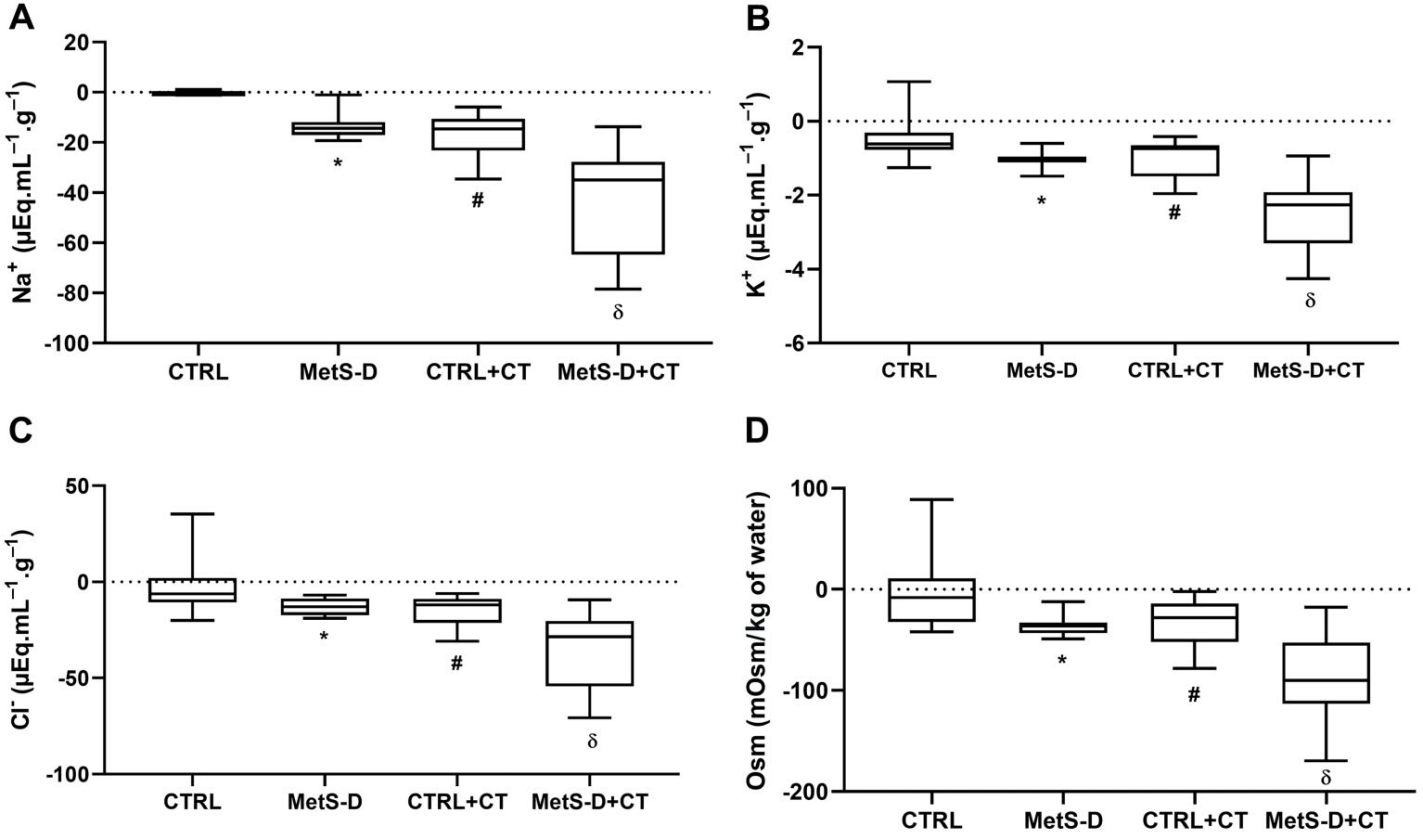
Impact of the diabetic-metabolic syndrome (MetS-D) on the intestinal hydroelectrolytic transporters. Transport gradient of (**A**) Na^+^; (**B**) K^+^; (**C**) Cl^-^; and (**D**) osmolarity (Osm) in the distal intestinal portion of animals in the control (CTRL), MetS-D, control + cholera toxin (CTRL+CT), and MetS-D + cholera toxin (MetS-D+CT) groups (n=6). Data are from day 14 of the experiment and are reported as median and Q1-Q3. *P<0.05, MetS-D *vs* CTRL, ^#^P<0.05 CTRL+CT *vs* CTRL, ^δ^P<0.01, MetS-D+CT *vs* MetS-D, Mann-Whitney test.

MetS-D animals had altered hydroelectrolytic absorption in the ileal portion. Negative values indicated excretion of the analyzed parameters. Thus, a significant increase (P<0.001) was observed in the excretion of sodium, potassium, and chloride in the MetS-D group compared to the control group (Figure 6A, B, and C, respectively). Osmolarity was also significantly increased in MetS-D animals (P<0.001; Figure 6D). Ileal perfusion of MetS-D animals revealed a secretory diarrheal condition. No significant difference (P>0.05) was observed between the MetS-D group and the CT control group for Na^+^, K^+^, Cl^-^, and osmolarity, indicating that the MetS-D generated an electrolyte and water secretory process similar to that of secretory diarrhea caused by CT. When diarrhea was induced in MetS-D animals, sodium values were 303.7% higher (P<0.0001) in the luminal content of MetS-D+CT compared to MetS-D animals (Figure 6A). Potassium was 144% higher (P<0.0001) in MetS-D+CT than in MetS-D animals (Figure 6B). Chloride and water excretion was also higher in diabetic animals with CT compared to MetS-D animals (Cl^-^: MetS-D -11.97 *vs* MetS-D+CT -28.58, P<0.0001; Osm: MetS-D −36.26 *vs* MetS-D+CT -90.13; P=0.0003) (Figure 6C and D, respectively).

### MetS-D altered the gene transcription of tight junctions

**Figure 7:**
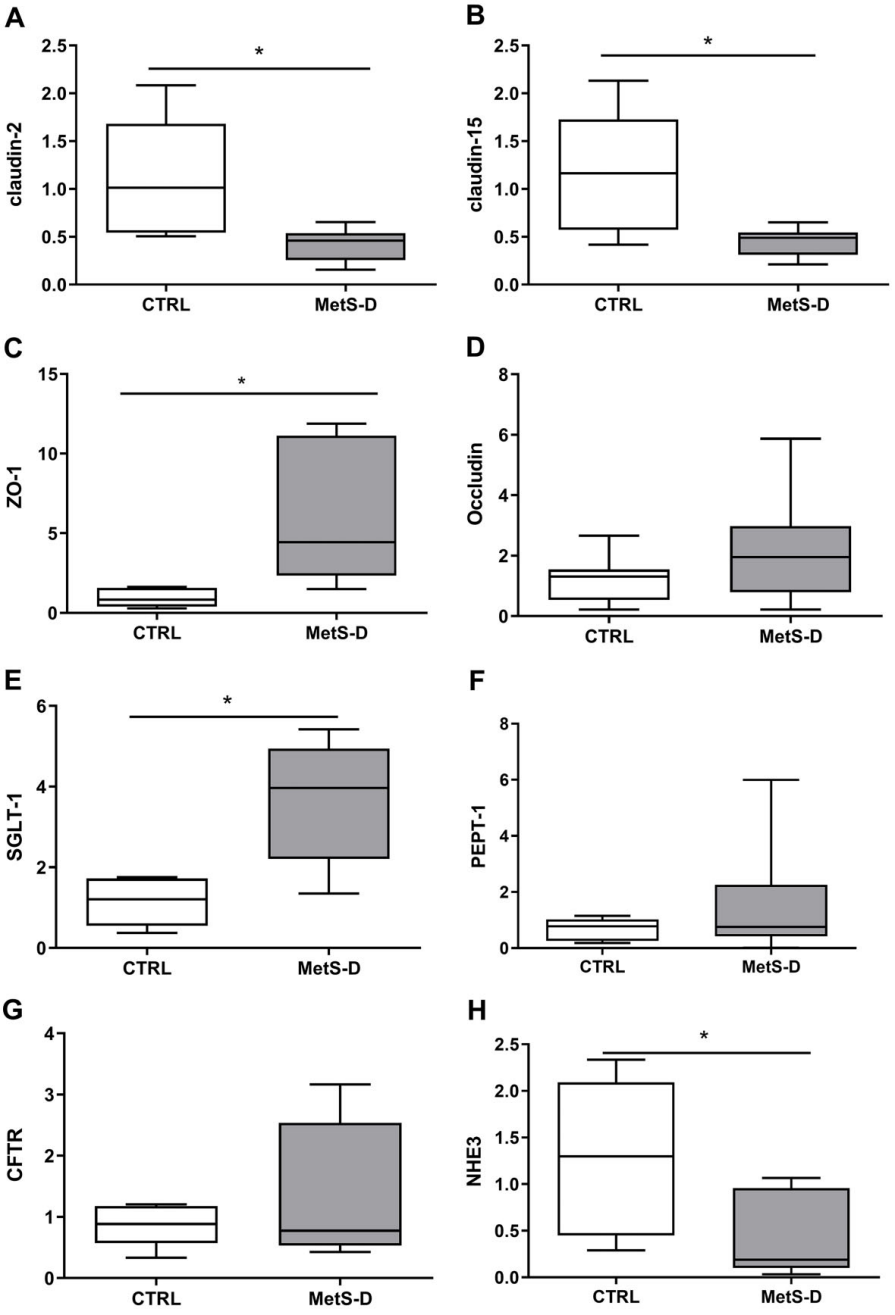
Relative transcription of genes for tight junctions, intestinal transporters, and ion channels regulatory proteins in the ileum of diabetic-metabolic syndrome (MetS-D) rats and controls (CTRL). Relative transcription of (**A**) claudin-2; (**B**) claudin-15; (**C**) zonulin-1 (ZO-1); (**D**) occludin; (**E**) SGLT-1; (**F**) PEPT-1; (**G**) CFTR; and (**H**) NHE3 in the ileum of MetS-D and CTRL groups (n=6-10). Data are reported as median and Q1-Q3. *P<0.05, Mann-Whitney test.

MetS-D animals characterized by high hyperglycemia generated a significant reduction in tissue claudin-2 and -15 mRNA expression (P<0.05; Figure 7A and B, respectively). In contrast, ZO-1 transcription was significantly increased in MetS-D animals (P<0.05; Figure 7C). In addition to modulating the gene transcription of tight junction proteins, MetS-D also altered the mRNAs of transcellular transporters, elevating SGLT-1 and reducing NHE3 gene expression (P<0.05; Figure 7E and H, respectively). No significant change (P>0.05) was observed for the other intestinal transcripts (occludin, PEPT-1, and CFTR; Figure 7D, F, and G).

## Discussion

In this study, MetS-D was characterized as a malnutrition status and critical metabolic disorder. Hyperglycemia was associated with a reduction in body weight, polyuria, polydipsia, a decrease in total body water and in intracellular and extracellular fluid, as well as disturbance in amino acid metabolism. Diabetes mellitus is a common condition in clinical routine, but it has varied, complex, and sometimes unknown symptoms (17). The present set of experimental evidence is characteristic of the clinical manifestations of diabetes, with model consistency validation (18).

Although MetS-D presents as an acute model of metabolic dysfunction, the present data indicated that metabolic disruption led to progression of responses in both type 1 and type 2 diabetes; etiologically, in the face of high glucose, it causes functional damage to organs and systems. This phenomenon still has limited understanding and unknown complex mechanisms (19). However, our experimental model can help in the experimental understanding of the evolution of hyperglycemic disorders in this pathophysiology.

In parallel, which became evident in the metabolic disruption, diabetic dysfunction also had an impact on the morphofunctional aspects of the gut epithelium, altering the villus and crypt area of the small intestine, the molecular and structural conduction linked to compensatory polyphagic phenomenon, and the inflammatory status in the animal model. The present data indicated an increase in intestinal permeability and disturbance in the functioning of the intestinal transporters of the diabetic rats. The pathophysiological events of MetS-D on the intestinal morphofunctional barrier were detailed in the context of severe hyperglycemia. In the present model, this condition triggered deviations in metabolic pathways, reflecting in clinical macro-observations such as weight loss, polyphagia, and changes in body composition.

The impact of diabetic disorders affects the structure of body tissues, mainly altering the energy reserves linked to protein and lipid metabolism. Specifically, MetS-D caused a critical change in body composition. Diabetes induces considerable changes in the body composition of patients, with a decline of body indices in relation to healthy individuals (20). The distinction of body percentages is linked to the imbalance of cellular fluids, which is also the basis for polydipsia and polyphagia of MetS-D animals.

The deficit in cellular energy considerably affected protein metabolism in MetS-D animals. The deterioration of this system modified serum essential amino acids. The systemic disposition of some important amino acids, such as glutamine and glutamic acid, was altered in diabetic animals. This event appears to be a compensatory mechanism for the progression of decompensated diabetes (21). In particular, alanine metabolism may play an important role in the diabetic syndrome. In animals with diabetic metabolic alteration, alanine concentration is reduced while skeletal muscle atrophy occurs (22). L-alanine supplementation protects against some organic dysfunction in diabetes, alleviating diabetic toxicity, restoring metabolic rates, and diminishing the effects of reactive oxygen species (ROS) deterioration (23).

Diabetic hyperglycemia reduces antioxidant defenses and elevates free radical levels, increasing cellular oxide status in the diabetic environment. The present data pointed to an imbalance between oxidative damage and antioxidant defense, confirmed by MDA and GSH levels in the intestinal tissue. Several studies have reported the correlation between hyperglycemia and the increase of ROS in the gut of T1D and T2DM models. This phenomenon is related to insulin resistance, presence of inflammation, and morphological disruption in the duodenum, jejunum, and ileum (24,25). The present findings are in agreement with the observations seen in clinical studies (26) of these disorders.

Parallel to the decrease in antioxidant biomarkers (GSH and alanine), the MetS-D group also had inflammation in the intestinal epithelium. MPO indicates the genesis and prognosis of metabolic disturbance in morbid patients with high serum glucose (27). Metabolic deviations modify the cellular parameters of specific organs. These changes are dependent on local oxidative and immunoinflammatory perturbation.

The present study indicated the molecular correlation between inflammation and ROS in the small intestine of MetS-D animals. This set of findings was related to the increase of intestinal permeability, as observed by the greater permeation of lactulose in diabetic animals. The intestinal absorption area was reduced in the MetS-D group as detected by morphometry.

Biomarkers of leaky gut syndrome in a diabetic preclinical model are still insufficient for detecting IEBF disruption. Here, we used the classical test for actulose and mannitol detection to detect the critical impairment of the IEBF. The rapid increase in paracellular permeation is associated with malnutrition and absorptive disorder, which may help to explain the compromised body composition of the MetS-D group. In the clinical setting, increased intestinal permeability is reported in individuals with T1D and metabolic syndrome (28). In these patients, the presence of inflammation and disruption in the intestinal microbiota are indicated as the cause of this damage (29,30).

The injury caused to IEBF by hyperglycemia seems to be dependent on dysbiosis and inflammation in the intestinal epithelium (31), which partly corroborates our findings of the MetS-D model, leaving the mechanisms related to the microbiota as future avenues of investigation.

It is known that changes in the configuration of the IEBF channels that form the paracellular route in tight junctions considerably affect the transport of glucose, amino acids, and bile acid, in addition to ionic gradients. Thus, a common manifestation in diabetic metabolic disorder is the presence of diarrhea. Although the methodological assay of the manifestation of diarrhea was not performed, the present study raised the hypothesis that Mets-D could contribute to a hydroelectrolyte disorder in the epithelium, leading to a diarrheal event. The consequences of hyperglycemia in a model of secretory diarrhea were previously analyzed (32). This pathophysiological model is the gold standard for analyzing the relationship between intestinal inflammation and secretory effect on the intestinal epithelium, showing an imbalance in transcellular transport in the gut epithelium, which can have an effect similar to the intestinal disruption due to diabetic hyperglycemia.

The pathobiology of secretory diarrhea in the CT model is complex, involving changes in apical transport and thus causing a critical hydroelectrolyte disturbance (33). Our data point to a considerable deficit in the homeostasis of the ionic transport of Na, K^+^, and Cl^-^, as well as a worsening in the maintenance of osmolar indexes. The MetS-D model had damage similar to the CT model, indicating a strong disturbance in electrolyte transport. Our model showed, for the first time, the similarity of the effects between the disruption of transcellular transport in the intestinal epithelium by a metabolic syndrome and those by an infectious agent that specifically acts in the absorptive function of the enterocyte. The presence of metabolic disturbance doubled the hydroelectrolyte transport, which may contribute to the pathobiology responses of the MetS-D model.

It was recently reported that diabetic diarrhea has a long duration, is painless, voluminous, and watery, and is most commonly found in long-standing, insulin-dependent patients with poor glycemic control (34). Our data corroborated this condition, and although it was an acute model, important molecular points of this phenomenon were indicated.

The increase in intestinal permeability was linked to considerable changes in the gene expression of transcellular transporters in the jejunal epithelium. Sensitive structural and functional alterations of cell tight junctions promote critical functional changes in the transcellular absorptive events of the intestinal epithelium; these changes were associated with serious malnutrition, impaired development, and early mortality in a preclinical model (9).

The present results showed a strong association between disruption of tight junctions and high paracellular permeation, a phenomenon dependent on the gene expression of claudin-2 and -15. These junctional proteins generate refined control of fluid and electrolyte flow, being essential for sodium homeostasis and absorption of glucose and amino acids (35). It has been suggested that morphofunctional alterations in the arrangement of claudin proteins contribute to the disruption of the paracellular pathway in T1D (1).

Maintenance of the integrity of the IEBF occurs through the interaction of claudin junctional proteins and intracellular ZO-1, ZO-2, and ZO-3. In contrast to the detection of claudin levels, there was an increase in ZO-1 gene transcription in the MetS-D group. Electrostatic interactions between these proteins are necessary for the functional-structural selectivity of tight junctions (9). The intracellular inflammatory process leads to a disruption in this interaction system, marked by the presence of cytokines such as tumor necrosis factor (TNF) and interferon (IFN)γ in intestinal epithelial cells, which contributes to the leaky gut syndrome in the intestine (36,37). Thus, it can be suggested that the elevation of ZO-1 in the MetS-D model may be a modulatory mechanism. The present study documented, for the first time, a molecular gene disarray between junctional (claudin-2 and -15) and intracellular markers (ZO-1) in the diabetic intestine, pointing to the need for more robust assays to understand this phenomenon.

During the progression of diabetes, there was an increased mRNA expression of the SGLT-1 transporter in the intestinal epithelium of the MetS-D group. Increase of SGLT-1 was reported in a preclinical study of T1D (38). Here the role of this protein in the diabetic syndrome was associated with a possible compensatory mechanism, which is not fully understood. Randomized clinical studies have shown that the inhibition of this transporter by a specific inhibitor improves glycemia and other clinical parameters, indicating this receptor as having a strong therapeutic potential for MetS-D (39). Thus, it has become a potential clinical target.

In our model, MetS-D affected NHE3 exchanger levels. The decreased expression of NHE3 is associated with aberrant fluid absorption in diabetes, and the restoration of fluid absorption involves a coordinated assembly of multiprotein complexes (40). A functional dysfunction in the NHE3 exchanger was correlated with the hydroelectrolyte disturbance detected. Changes at the molecular level contribute to structural changes in the small intestine in diabetic syndrome (4). This impairment of intestinal epithelial structures can lead to serious complications, such as malnutrition and duodenal ulcers in diabetics.

This work was limited by the lack of assessment of protein expression of the intestinal transporters that form tight junctions to better understand the modulations in the IEBF during an acute phase of hyperglycemia. The analysis of the glucose and amino acid transport in Üssing chambers would be important to better understand the transcellular transport association of macromolecules under MetS-D conditions. Furthermore, as differences in microbiota composition affect MetS-D, a microbiota analysis in this model would indicate the altered taxa in the acute phase. However, this study provided unprecedented evidence of a complex regulation of tight junction forming proteins and intestinal transporters, which culminate in increased paracellular permeability and a secretory state in the IEBF response to MetS-D. We proposed an innovative experimental model of pathophysiological phenomena of diabetic metabolic dysfunction associated with intestinal disturbance not yet studied in detail in preclinical models.

In conclusion, the set of findings indicated that MetS-D critically affected metabolic routes, while also having an impact on the structural aspects of the intestine affecting the villus and crypt area, leading to increased intestinal permeability and considerable disorder in the functioning of intestinal transporters located in the epithelial cells of the small intestine.

## Supplementary Material

Click here to view [pdf].
