# Supplementary material for: Induced acute hyperglycemia modifies the barrier function of the intestinal epithelium by tissue inflammation and tight junction disruption resulting in hydroelectrolytic secretion in an animal model
Source: Braz J Med Biol Res. 2024 Apr 19;57:e13309. doi: 10.1590/1414-431X2024e13309 (PMC11027184; doi:10.1590/1414-431X2024e13309)

Supplementary material

Table S1. Gene primers and annealing condition.

Genes	Sequence of primers	Temperature
Occludin	S – AAC AGC CCC CTA ATG TGG AAG<br>AS – GAG TAG GCC ATT GGA ACT GTCG	60ºC
ZO-1	S – CTC GCA CGT ATC ACA AGC TGA<br>AS – CCT CAG GAT ATG GCT CCT TCC	60ºC
Claudin-2	S – AGG ACT TCC TGC TGA CAT CCA<br>AS – TCC ACC CAC TAC AGC CAC TCT	60ºC
Claudin-15	S – GAC CTC TCC AGA AAG GCC AAG<br>AS – AGA TAG CAA CCA ATG CCA CAGG	58ºC
NHE3	S – CTC TGG GGC AGG AAT TGA TA<br>AS – AGA TAG CAA CCA TGC CAC AAG	58ºC
CFTR	S – GCA ATG GGC TGT GAA CTC AA<br>AS – ACC TCC AGA GGG CCA GGT AT	60ºC
SGLT-1	S – GCT GGA GTC TAC GTA ACA GCA CA<br>AS – GGG CTT CTG CAT CTA TTT CAA TG	58ºC
PEPT-1	S – AGC AGA GAT CGA GGC ACA GT<br>AS – TTC CCT ACG CCC TTT TTC TT	60ºC
GAPDH	S – GTT ACC AGG GC TGCC TTC TCT<br>AS – AAC TTG CCG TGG GTA GAG TCA	60ºC

S: sense primer (5'-3'); AS: antisense primer (3'-5'); reference gene: GAPDH.

Figure S1. Serum albumin levels and nitrogen balance in MetS-D. **A**, Serum albumin levels and nitrogen balance values in MetS-D and control animals (n= 6–10). **B**, Nitrogen balance values correspond to ingested nitrogen (g protein/6.25) subtracted from excreted nitrogen (24h urea g x 0.47). Data are reported as mean ± SE.*P<0.05; unpaired data, Student's *t*-test.

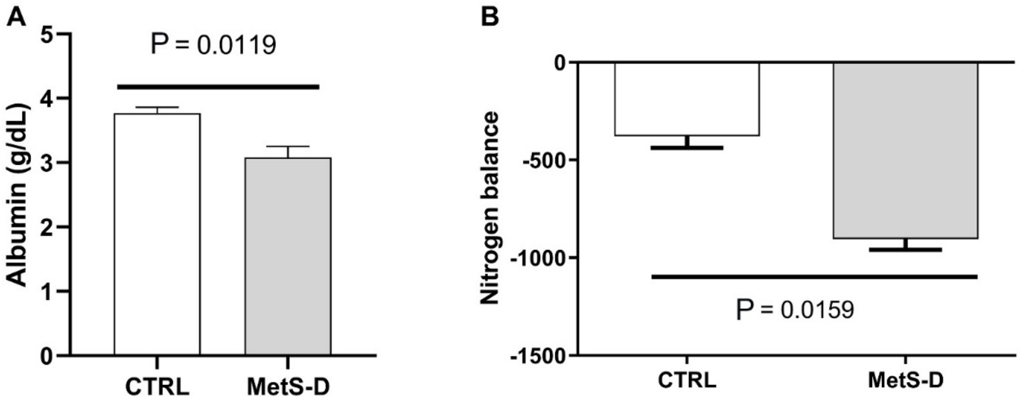

Supplement: Supplementary file 1 [file 1414-431X-bjmbr-57-e13309-suppl.pdf]
